# Investigations of Structural, Magnetic, and Electrochemical Properties of NiFe_2_O_4_ Nanoparticles as Electrode Materials for Supercapacitor Applications

**DOI:** 10.3390/ma16124328

**Published:** 2023-06-12

**Authors:** Shalendra Kumar, Faheem Ahmed, Nagih M. Shaalan, Nishat Arshi, Saurabh Dalela, Keun Hwa Chae

**Affiliations:** 1 Department of Physics, College of Science, King Faisal University, P.O. Box 400, Al-Ahsa 31982, Saudi Arabia; 2 Department of Physics, University of Petroleum & Energy Studies, Dehradun 248007, India; 3 Physics Department, Faculty of Science, Assiut University, Assiut 71516, Egypt; 4 Department of Basic Sciences, Preparatory Year Deanship, King Faisal University, P.O. Box 400, Al-Ahsa 31982, Saudi Arabia; 5 Department of Pure & Applied Physics, University of Kota, Kota, Rajasthan 324005, India; 6 Advanced Analysis & Data Center, Korea Institute of Science and Technology, Seoul 02792, Republic of Korea

**Keywords:** NiFe_2_O_4_ nanoparticles, dielectric, modulus, AC conductivity, ferromagnetism, supercapacitor

## Abstract

Magnetic nanoparticles of NiFe_2_O_4_ were successfully prepared by utilizing the sol–gel techniques. The prepared samples were investigated through various techniques such as X-ray diffraction (XRD), transmission electron microscopy (TEM), dielectric spectroscopy, DC magnetization and electrochemical measurements. XRD data analysed using Rietveld refinement procedure inferred that NiFe_2_O_4_ nanoparticles displayed a single-phase nature with face-centred cubic crystallinity with space group Fd-3m. Average crystallite size estimated using the XRD patterns was observed to be ~10 nm. The ring pattern observed in the selected area electron diffraction pattern (SAED) also confirmed the single-phase formation in NiFe_2_O_4_ nanoparticles. TEM micrographs confirmed the uniformly distributed nanoparticles with spherical shape and an average particle size of 9.7 nm. Raman spectroscopy showed characteristic bands corresponding to NiFe_2_O_4_ with a shift of the A_1g_ mode, which may be due to possible development of oxygen vacancies. Dielectric constant, measured at different temperatures, increased with temperature and decreased with increase in frequency at all temperatures. The Havrilliak–Negami model used to study the dielectric spectroscopy indicated that a NiFe_2_O_4_ nanoparticles display non-Debye type relaxation. Jonscher’s power law was utilized for the calculation of the exponent and DC conductivity. The exponent values clearly demonstrated the non-ohmic behaviour of NiFe_2_O_4_ nanoparticles. The dielectric constant of the nanoparticles was found to be >300, showing a normal dispersive behaviour. AC conductivity showed an increase with the rise in temperature with the highest value of 3.4 × 10^−9^ S/cm at 323 K. The M-H curves revealed the ferromagnetic behaviour of a NiFe_2_O_4_ nanoparticle. The ZFC and FC studies suggested a blocking temperature of ~64 K. The saturation of magnetization determined using the law of approach to saturation was ~61.4 emu/g at 10 K, corresponding to the magnetic anisotropy ~2.9 × 10^4^ erg/cm^3^. Electrochemical studies showed that a specific capacitance of ~600 F g^−1^ was observed from the cyclic voltammetry and galvanostatic charge–discharge, which suggested its utilization as a potential electrode for supercapacitor applications.

## 1. Introduction

Addressing the demand of the increasing population for the highly efficient systems of energy storage and harvesting have led the scientific community to search for device fabrication with materials offering enhanced capacity and durability [1,2,3,4,5]. The production of devices demonstrating high energy and power densities is in great demand. The utilization of electrochemical reactions for the energy storage and harvesting is a promising technique. Therefore, the fabrication of supercapacitors based on electrochemical reactions have gained significant attention of researchers. The performance of the supercapacitor relies upon the capacitive performance, long cycle life, less temperature-sensitivity and electrochemical stability of the material used for making the electrode. The parameters that measure the performance of a supercapacitor are specific capacitance, rate of charging/discharging cycles, cyclic stability, capacity retention rate, efficiency, energy density and power density. Interestingly, metal oxides exhibit virtuous redox properties as well as low resistance [6]. It is observed that the spinel ferrites with AB_2_O_4_ structure, formed with varying cation distribution, have procured a significant position in search of suitable materials for supercapacitor fabrication attributable to their excellent structural and physical/chemical properties. NiFe_2_O_4_ is the type of metal oxide with inverse spinel structure that exhibits highly desirable stable electrochemical behaviour [7,8,9]. In this symmetry, Fe ions occupying tetrahedral site may be in a mixed valence state, i.e., Fe^2+/3+^ which provides the redox characteristics and may facilitate the exchange interactions and the carrier hopping mechanism. The electrochemical properties of NiFe_2_O_4_ nanoparticles have been utilized for the sensing applications of electrochemical detection of nitrite as studied by Nithyayini et al. [10]. Furthermore, NiFe_2_O_4_ is environmentally friendly and abundant in nature. Therefore, the performance of electrode material prepared by NiFe_2_O_4_ has been investigated by various research groups [5,11,12,13,14,15,16,17]. The chemically synthesized NiFe_2_O_4_, investigated previously have shown a specific capacitance ~120 F g^−1^ measured with a two-electrode configuration [18]. The mesoporous NiFe_2_O_4_ prepared via hydrothermal synthesis has been investigated by Kumar et al. for its electrochemical performance. They examined the electrochemical response of the synthesized nanoparticles with a high surface area in a three-electrode configuration cell [17]. Ferrite/graphene nanocomposite electrodes were prepared by Saom et al., who studied their performance using electrochemical measurements. The authors observed the specific capacitance value of ~207 F g^−1^ in a 1 M Na_2_SO_4_ electrolyte [19]. They suggested that the improvement in the specific capacitance is about four times greater than that in the NiFe_2_O_4_ electrode.

The specific capacitance of NiFe_2_O_4_ can be improved over a number of cycles. For instance, Gao et al. have detected enhancement in the value of specific capacitance of as much as 128% after 2000 cycles. They reported an energy density of ~10.15 Wh/kg and a power density of ~140 W/kg [20]. In another work, the NiFe_2_O_4_ nanospheres prepared by Arun et al. reported 277 F g^−1^ of specific capacitance with a 101% capacitance retention after up to 5000 cycles. The authors reported that the reduction in particle size increased the capacity retention up to 126% at 5000 cycles [21]. Thus, the variation in crystal structure, the nature of dopants and morphology are the primary factors to enhance the electrochemical performance of a material [22,23]. However, the challenge faced by the researchers in improving the electrochemical performance of NiFe_2_O_4_ is its low electrical conductivity. Nonetheless, the electrochemical performance of NiFe_2_O_4_ is tunable due to its variable cation distributions among the sublattice of the spinel structure as well as its morphology. In this research work, the chemical method was utilized for the preparation of NiFe_2_O_4_ nanoparticles. As-synthesized NiFe_2_O_4_ nanoparticles were characterized using numerous techniques. The structural, morphological and magnetic properties were investigated using X-ray diffraction, transmission electron microscopy and DC magnetization measurements. The electrochemical properties studied through cyclic voltammetry, galvanostatic charge–discharge cycling and electrochemical impedance spectroscopy indicated the improved performance of a NiFe_2_O_4_ electrode which can be utilized as potential anode material for energy storage applications.

## 2. Experimental Details

The chemicals used for the typical synthesis of NiFe_2_O_4_ were iron (III) nitrate nonahydrate, nickel (II) nitrate hexahydrate, citric acid, ethylene glycol, purchased from CDH: central drug house, India. To synthesize Ni–Ferrite, citrate gel (sol–gel) method was followed. First, citric acid was mixed in DI water (50 mL) to prepare a solvent (0.1 M). The solution was stirred magnetically at room temperature to produce a homogeneous solution. After the homogeneous mixing, the stoichiometric amount of Fe(NO_3_)_3_·9H_2_O was added in the solvent. Then, 209 μL of Conc. HNO_3_ was poured while continuously stirring to completely dissolve the components of the solution. After 1 h, Ni(NO_3_)_2_·6H_2_O was transferred to the solution by maintaining the Ni^2+^/Fe^3+^ ratio at 0.5. The stirring was continued for another 1 h. Then, 0.577 mL of ethylene glycol was added for the formation of the gel. After the ethylene glycol was added, the heating was started (80 °C) while continuously stirring until the formation of gel. The gel was allowed to go under combustion at the same temperature. Once the gel was dried, it was grinded using pastel mortar preceding the formation of a fine powder. The powder was finally heat treated at 600 °C for 5 h in an open box furnace to obtain the final product. The as-prepared nanoparticles were studied utilizing numerous techniques such as x-ray diffraction (XRD), transmission electron microscopy (TEM), dielectric spectroscopy, DC magnetization and cyclic voltammetry (CV) measurements. The XRD spectrum was recorded in a *θ*–2*θ* mode using Bruker D8 advanced diffractometer in the range of 20–80° (0.02 deg/s) (λ Cu-Kα = 0.15418 nm). The microstructural properties and the selected area electron diffraction (SAED) pattern of the as-prepared nanoparticles was investigated using transmission electron microscope (JEOL, Model-2100, Corporation Place, Singapore). DC magnetization measurements were conducted using the Quantum Design physical property measurement setup PPMS-6000 (Quantum design, manufacturer, San Diego, CA, USA) at different temperature. Alpha-A high-performance frequency analyser (Novocontrol technologies GmbH & Co.KG, Montabaur, Gemany) was employed for temperature-dependent dielectric spectroscopy measurements. In order to record the dielectric spectroscopy data, the NiFe_2_O_4_ powder was pressed in the form of a circular (diameter 10.0 mm and thickness 1.25 mm) pallet by applying a pressure of 5.0 ton using hydraulic pressure machine. For electrical measurements, both faces of the pallets were coated with the conductive silver paste. Dielectric spectroscopy was recorded in the frequency of 1.0 Hz to 10.0 MHz at various temperatures. The real (*ε*′) and imaginary (*ε*″) part dielectric constant was calculated using [24]: $\varepsilon^{\prime }=\frac{C\;\times \;t}{\varepsilon_{0}\;\times \;A}$ and $\varepsilon^{\prime \prime }=\varepsilon^{\prime }tan\delta$, respectively, where *C* denotes the capacitance, *A* and *t* represent the area and sample thickness, whereas $\varepsilon_{0}$ and tanδ signify the permittivity of the free space and dielectric loss tangent. The frequency-dependent AC conductivity (⌠_ac_) can be calculated using formula [24], $\sigma_{ac}=2\pi f\varepsilon_{0}\varepsilon^{\prime \prime }$, where f is frequency. A Corrtest-CS150 electrochemical workstation in a typical three-electrode configuration was utilized for galvanostatic charge–discharge (GCD), cyclic voltammetry (CV), as well as electrochemical impedance spectroscopy (EIS). Electrochemical studies were performed in a 1.0 M Na_2_SO_4_ electrolyte along with Ag/AgCl as a reference electrode and Pt as a counter electrode, respectively. The EIS data were obtained in the frequency range of 1 Hz–100 kHz.

## 3. Results and Discussion

### 3.1. Structural Analysis

**Figure 1:**
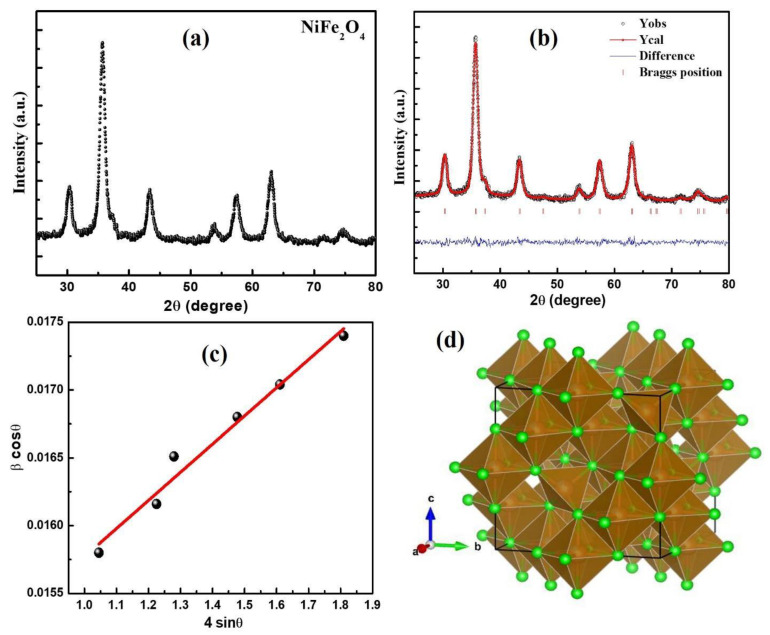
(**a**) Experimentally obtained X-ray diffraction pattern; (**b**) Rietveld refinement of NiFe_2_O_4_; black data points indicate the experimental curve, red line indicates the theoretically fitted curve, vertical pink line indicates Bragg’s positions and blue line at the bottom indicates the difference between the experimental curve; (**c**) Williamson–Hall plot; (**d**) crystal structure obtained from the Rietveld refined pattern.

The XRD pattern corresponding to NiFe_2_O_4_ nanoparticles measured in θ–2θ at room temperature is illustrated in Figure 1a. The experimentally observed diffraction pattern is in accordance with the standard data file JCPDS No. 074-2081 [25], which corresponds to the single-phase cubic symmetry of the structure with a space group Fd-3m. Reflection planes (220), (311), (400), (422), (511) and (440) correspond to the inverse spinel structure and revealing polycrystalline nature of NiFe_2_O_4_ nanoparticles. The absence of extra peaks rules out the formation of any secondary phases. Furthermore, detailed structural analysis was carried out using Rietveld refinement of the XRD pattern. Figure 1b shows the Rietveld refined diffraction pattern of NiFe_2_O_4_ nanoparticles performed using the FULLPROF program. The experimental data (black colour circles) agrees well with the theoretical pattern (red line) with a small difference (blue colour line). The peak positions of the reflection planes are shown by the vertical lines in pink colour. The reflection planes in the diffraction pattern corresponds to the single-phase cubic symmetry of the structure with a space group Fd-3m. The various crystallographic parameters such as lattice constant, unit cell volume, crystallite dimension, lattice strain, and theoretical x-ray density were determined with the help of the XRD pattern. Lattice parameter comes out to be 8.34 Å with a cell volume of 580.18 Å^3^. Crystallite size (D) and strain (ε) were estimated from XRD diffractions peaks using Williamson Hall plot with the following expression:

*β cosθ* = *4ε sinθ* + *Kλ/D*, (1)

where *K* (~0.89) is the structure factor, *β* is the FWHM, 2*θ* is the Bragg’s angle and *λ* (~1.5046 Å) is the wavelength of the X-ray used. Equation (1) is represented in Figure 1c. The crystallite size is found to be 10 nm. The value of strain is observed to be 2.1 × 10^−3^. Further, the X-ray density (d_hkl_) of NiFe_2_O_4_ nanoparticles is calculated using formula d_hkl_ = 8 M/Na^3^, where a^3^, N and M represent the volume, Avogadro’s number and molecular weight of the sample, respectively. The value of the d_hkl_ is observed to be 5.37 g/cm^3^. The unit cell structure obtained from Rietveld refinement is shown in Figure 1d. Further, the dislocation density was calculated using 1/D^2^, *D* being the crystallite size which comes out to be ~10^−2^ m^−2^.

### 3.2. Morphological Analysis

**Figure 2:**
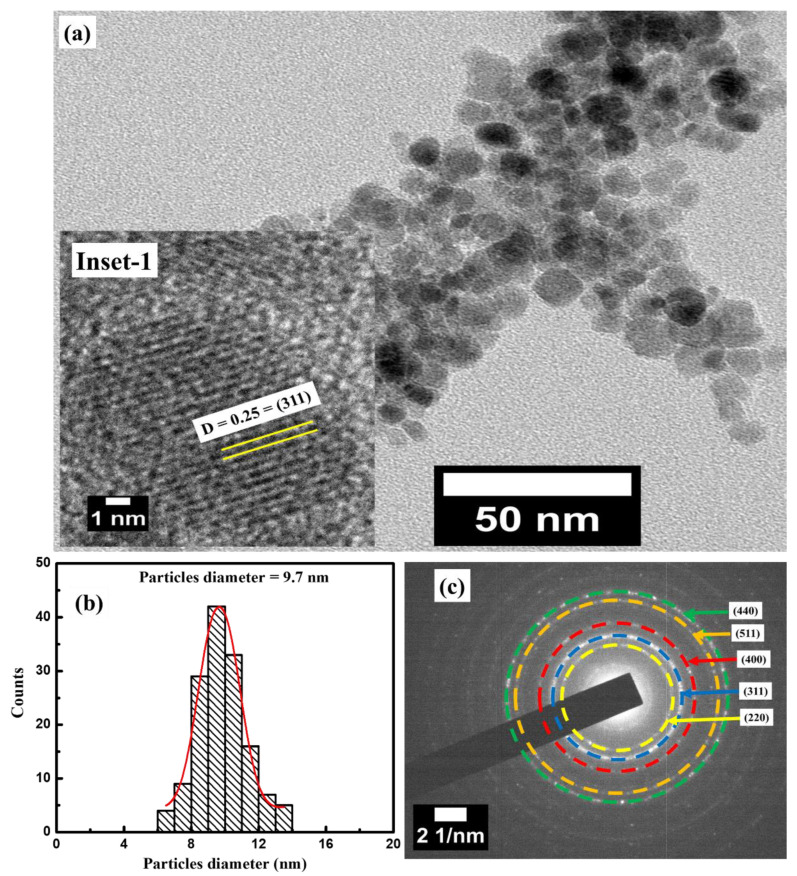
(**a**) TEM micrographs and inset-1 highlights HR-TEM micrograph; (**b**) size distribution histograms; (**c**) SAED patterns of NiFe_2_O_4_ nanoparticles; rings corresponding to different planes are highlighted in different colour.

The TEM and SAED measurements were conducted to investigate the microstructure and phase information of NiFe_2_O_4_ nanoparticles. Figure 2a indicates the TEM micrographs of the as-prepared sample. The micrographs show the uniformly distributed spherical shape morphology of the nanoparticles. The average particle size of ~10 nm is observed from size distribution histogram as shown in Figure 2b, which displays a narrow range distribution in agreement with uniform morphology distribution (Figure 2a). Furthermore, inset-1 shows the HR-TEM image of NiFe_2_O_4_ nanoparticles which is utilized to calculate the interplanar distance (D). The value of the D measured using HR-TEM micrograph was found to be 0.25 nm, which corresponds to the (311) plane of the FCC crystal structure. The SAED pattern of NiFe_2_O_4_ nanoparticles is displayed in Figure 2c. The various rings observed in the SAED pattern correspond to (220), (311), (400), (511), and (440), which indicate the reflections agreeing to the Fd-3m cubic symmetry.

### 3.3. Raman Spectroscopy

**Figure 3:**
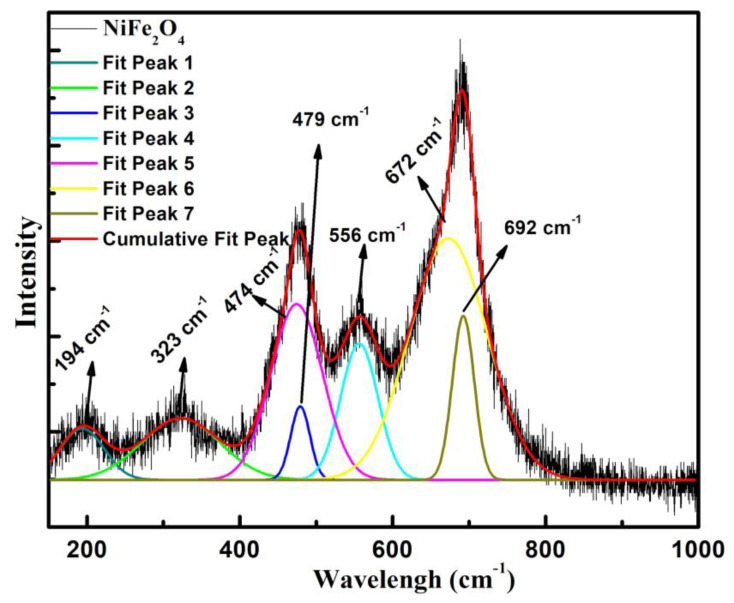
Raman spectra of NiFe_2_O_4_ nanoparticles prepared using citrate sol-gel method.

The results of Raman spectroscopy, a non-destructive technique, on NiFe_2_O_4_ nanoparticles is displayed in Figure 3, obtained in 175–1000 cm^−1^ as an important probe for structural properties. The spectra reveal five Raman bands corresponding to the inverse spinel structure of NiFe_2_O_4_ with three active bands: A_1g_, E_g_, 3T_2g_. Out of these bands, A_1g_ emerges as a consequence of symmetrical stretching of metal–oxygen bands, appearing as the strongest mode at ~690 cm^−1^. On the other hand, Eg indicated the symmetric bending of oxygen in respect to cations at tetrahedral sites, appearing at about 320 cm^−1^. The A_1g_ and E_g_ bands involve metals present at tetrahedral sites in the NiFe_2_O_4_ matric. Out of three T_2g_ bands; T_2g_(I) and T_2g_(II) represent the asymmetric bending and stretching of the metal–oxygen band with metals at octahedral sites, whereas T_2g_(III) is attributable to the translational movement of the metal–oxygen bond involving metals at tetrahedral sites [26]. The maximum intensity band, obtained at 692 cm^−1^, is associated with A_1g_. The E_g_ band was observed at 323 cm^−1^, whereas the three bands attributable to T_2g_ were observed at 194, 479 and 556 cm^−1^, respectively. The shoulders towards the left of the bands give the appearance of doublet-like features [27]. These bands are in agreement with the previously reported results [28]. These doublet-like features providing broadness to the bands are attributable to the distribution of Ni^2+^ and Fe^3+^ ions in the tetrahedra MO_4_ and octahedra MO_6_. This distribution indicates Ni^2+^ in the Ni^2+^-O_6_ octahedra, while, Fe^3+^ occupies the sites forming both the tetrahedra Fe^3+^-O_4_ and Fe^3+^-O_6_ [26]. Thus, the Raman spectra notifies the characteristic bands of NiFe_2_O_4_, and the result implies that the nanocrystalline nature of NiFe_2_O_4_ nanoparticles is uniform as reported by our XRD analysis. Further, the shift of A_1g_ band may also be observed in Raman spectra that could be caused by the development of oxygen vacancies in the lattice.

### 3.4. Dielectric Spectroscopy

**Figure 4:**
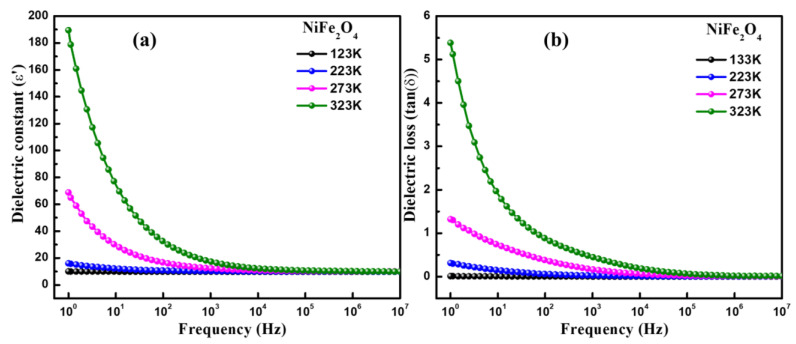
Comparative representation of (**a**) dielectric constant (*ε*′), and (**b**) loss tangent (tan*δ*) of NiFe_2_O_4_ as a function of frequency at different temperature.

Figure 4a shows the dielectric constant (*ε*′) vs frequency curves of NiFe_2_O_4_ measured at different temperatures. NiFe_2_O_4_ demonstrates the typical frequency-dependent behaviour showing dielectric dispersion. The dielectric constant is observed to increase with temperature as shown in Table 1. The comparative representation of dielectric constants at various temperatures is represented in Figure 4a. The increase in the dielectric constant with increasing temperature occurs due to the interfacial polarization which is significantly temperature-dependent and increases with temperature [29]. This phenomenon of interfacial polarization is explained in the following discussion. Higher *ε*′ at lower frequencies is found at the lowest frequency and decreases with increasing frequency, becoming steady at higher frequencies [30]. Dielectric constant behaviour can be understood by interfacial polarisation studied via a Maxwell–Wagner model [24]. The interfacial polarization is developed at the boundaries of the grains which are present in the material. The grain boundaries offer high resistance compared to the grains, which leads to the development of the polarization at the interfaces/boundaries. The high value of *ε*′ at low frequencies is attributable to the high resistance offered by grain boundaries. At lower frequencies, the influence of the grain boundaries is prominent, while at high frequencies, the resistance offered by grain is prominent. Thus, the space charge accumulation at the boundaries on applying voltage furnishes free charge carriers and leads to the development of interfacial polarization. The interfacial polarization in this way develops the dielectric dispersion through the medium. Interfacial polarization corroborates well with the Koop phenomenological theory [31]. Although the nature of dielectric dispersion may be ionic, dipolar or space charge, the process of dielectric dispersion in ferrites is analogous to the electrical conduction mainly on account of Fe^3+^/Fe^2+^ ions present at the octahedral sites. These ions undergo exchange of electrons through local displacement/hopping. The displacement occurs along the applied field and gives rise to polarization. The greater the electron hopping, the greater the polarization. At low frequencies, the amount of hopping of electrons among sites is large, corresponding to a high dielectric constant; however, the hopping of electrons reduces as the frequency increases due to the inability of the electrons to follow the frequency, which makes the dielectric polarization nearly consistent at higher frequencies [31,32]. The tan*δ* vs frequency (1.0 Hz to 10 MHz) measured at different temperatures is shown in Figure 4b. It can be seen that as applied frequency increases, the value of tan*δ* decreases, which signify a usual dispersion behaviour. However, it can be observed that tan*δ* increases in temperature as represented in Table 1. There are various reports in the literature that explain the reason for the decrease in the tan*δ* value with enhancing the frequency [24,26]. The value of tan*δ* demonstrates loss of energy, which dissipates in the form of heat on the application of the external field through the dielectric material. We further utilized the Havriliak–Negami (H–N) model for studying the characteristic relaxation and dielectric strength using the real as well as the imaginary part of the dielectric constant. The H–N model can be expressed using the following equation [29,33]:

(2) $$\varepsilon_{HN}^{*}\left(\omega \right)=\varepsilon^{\prime }\left(\omega \right)-i\varepsilon^{\prime \prime }\left(\omega \right)=\varepsilon_{\infty }+\frac{\varepsilon_{s}-\varepsilon_{\infty }}{{\left(1+{\left(i\omega \tau_{HN}\right)}^{\alpha }\right)}^{\beta }}+\frac{S}{i\omega^{p}},$$

where the real part of the dielectric is shown by

(3) $$\varepsilon^{\prime }\left(\omega \right)=\varepsilon_{\infty }+Re\left\lfloor \frac{\varepsilon_{s}-\varepsilon_{\infty }}{{\left(1+{\left(i\omega \tau_{HN}\right)}^{\alpha }\right)}^{\beta }}\right\rfloor$$

and the imaginary dielectric constant is represented via

(4) $$\varepsilon^{\prime \prime }\left(\omega \right)=Im\lfloor \frac{\varepsilon_{s}-\varepsilon_{\infty }}{{\left(1+{\left(i\omega \tau_{HN}\right)}^{\alpha }\right)}^{\beta }}\rfloor +\frac{S}{i\omega^{n}}.$$

**Figure 5:**
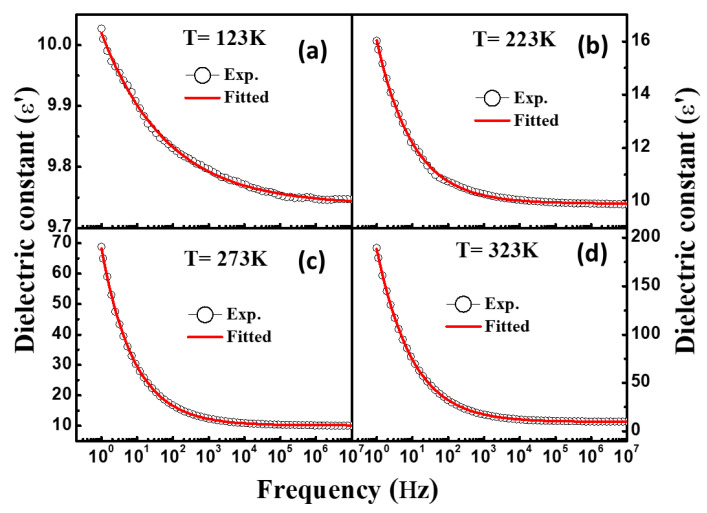
(**a**–**d**) Fitting of frequency-dependent dielectric constant (*ε*′) with Havriliak–Negami model at different temperatures.

**Figure 6:**
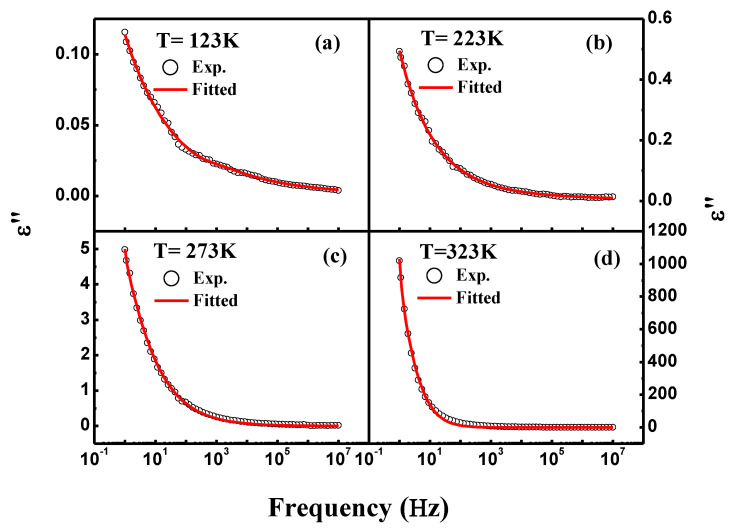
(**a**–**d**) Fitting of frequency-dependent dielectric loss (*ε*″) with Havriliak–Negami model at different temperatures.

Here, dielectric relaxation strength can be determined using formula $∆\varepsilon =\varepsilon_{s}-\varepsilon_{\infty }$, where $\varepsilon_{s}$ ($\varepsilon_{s}=\underset{\omega \tau_{\mathrm{HM}}\ll 1}{\lim}\varepsilon^{\prime }(\omega )$) denotes the relaxed permittivity while $\varepsilon_{\infty }$ ($\varepsilon_{\infty }=\underset{\omega \tau_{\mathrm{HN}}\ll 1}{\lim}\varepsilon^{\prime \prime }(\omega )$) represents the unrelaxed permittivity of the materials. Here, $\tau_{HN}$, signifies the characteristic relaxation time, and both *α* and *β* denote the fractional shape parameters. The values of *S* and n designate the DC conductivity and frequency exponent. Figure 5a–d and Figure 6a–d depict the real- and imaginary-part dielectric constant fitted using the H–N formalism at various temperatures. The temperature-dependent values of ∆ε, *α* and *β* calculated using the H–N model are displayed in Table 1. It was found that the sample showed the ideal Debye relaxation if the values of *α* and *β* were to be a unit, otherwise it demonstrated the non-Debye type relaxation. The values of *β* determined for NiFe_2_O_4_ nanoparticles were found in the range of 0.35 to 0.53, which clearly suggests the non-Debye type relaxation in the studied sample.

### 3.5. AC Conductivity

**Figure 7:**
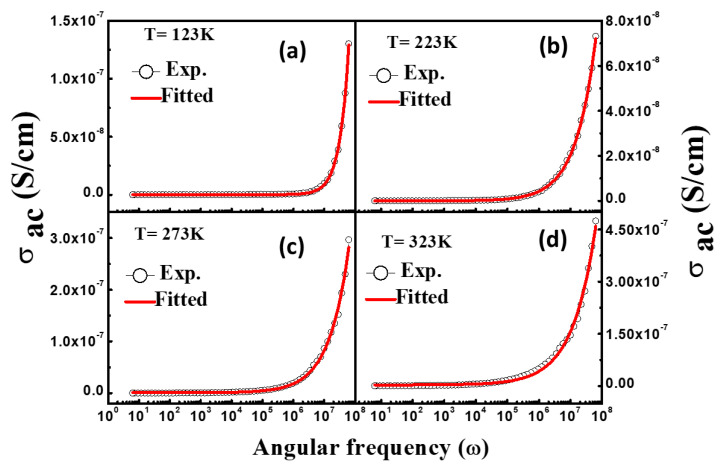
(**a**–**d**) AC conductivity of NiFe_2_O_4_ fitted using Jonscher’s power law.

The AC conductivity was calculated from the dielectric spectroscopy using equation *σ_ac_* = *ε* *ε*_0_*ω* tan *δ* [24] in 1 Hz–10 MHz of frequency range at different temperatures as observed in Figure 7a–d. From Figure 7a–d, it can be observed that the AC conductivity varies slowly at lower frequencies but shows a sharp rise in the high-frequency range. This is a usual trend exhibited by ferrites. The AC conductivity measurements indicated the highest value of 1.0 MHz at the temperature of 373 K. The low value of *σ_ac_* in the low-frequency state is owing to the fact that the electron exchange in dielectric materials needs high energy consumption; as a result of it, the exchange of electrons among Fe^2+^/Fe^3+^ exhibits high resistance. However, the energy required for this process at high frequency is delivered by the applied AC field, which decreases the resistance thus increasing the conductivity. In ferrite, there are two components: one is DC conductivity (frequency-independent) and other is AC conductivity (frequency-dependent) which contributes to electrical conductivity. More specifically, the conduction process in NiFe_2_O_4_ nanoparticles was studied using the Jonscher’s power law as given below:

(5) $$\sigma_{ac}=\sigma_{dc}+A\omega^{n},$$

where A represents the temperature-dependent constant, $\sigma_{dc}$ highlights DC conductivity, ω is angular frequency and *n* shows the exponent of the power law. Figure 7a–d illustrate the frequency-dependent AC conductivity of NiFe_2_O_4_ nanoparticles at different temperatures. The DC conductivity and exponent were calculated via Jonscher’s power law (see Table 1). The value of DC conductivity was found to be 1.1 × 10^−10^, 3.0 × 10^−10^, 8.3 × 10^−10^, and 3.4 × 10^−9^ at 123 K, 223 K, 273 K, and 323 K, respectively. The increase in DC conductivity with rising temperature corresponds to semiconducting behavior. It is worth mentioning that in the low-frequency region, the AC conductivity shows a frequency-independent plateau which is dominated by DC conductivity. This type of conduction is related to the grain boundaries which commonly dominate at low frequencies because of long-range ionic motion [34]. Yadav et al. explained that this type of behavior may also be associated with the p- and n-type carriers [35]. Furthermore, the values of frequency exponent (*n*) are estimated as 0.95, 0.70, 0.65 and 0.60 at 123 K, 223 K, 273 K, and 323 K, respectively. The exponent values decreased with rise in the temperature and deviated from the unity. The deviation of the exponent from the unity demonstrates non-ohmic behavior.

### 3.6. Modulus Spectroscopy

**Figure 8:**
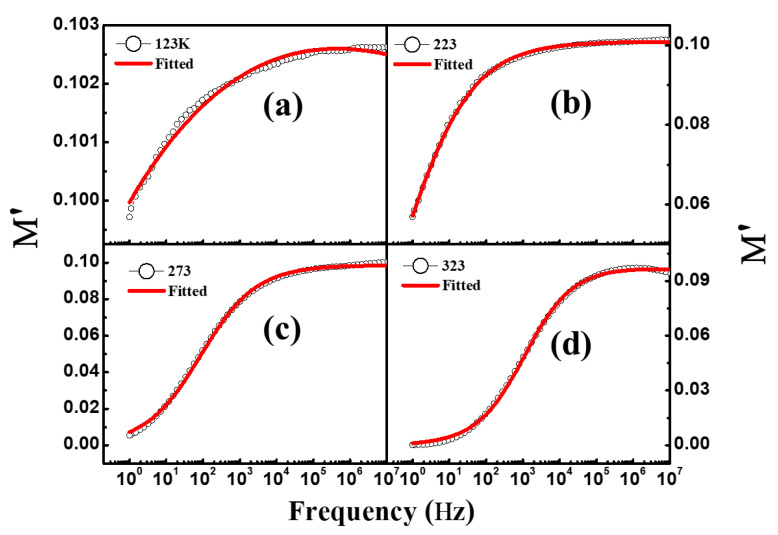
(**a**–**d**) Real part of modulus (*M′*) spectra of NiFe_2_O_4_ nanoparticles at different temperatures.

**Figure 9:**
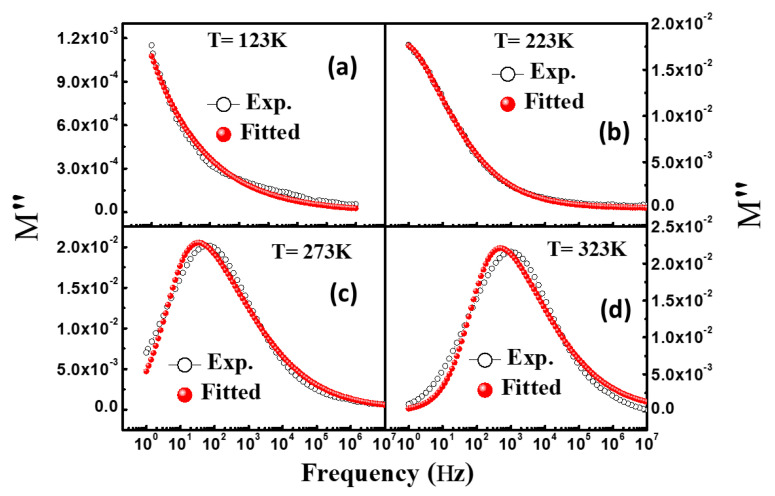
(**a**–**d**) Imaginary part of modulus (*M″*) spectra of NiFe_2_O_4_ nanoparticles at different temperatures.

Modulus spectroscopy is employed to understand the capacitive nature, conductivity mechanism, and relaxation phenomenon of dielectric materials [36] as it excludes the electrode polarization effect. The complex modulus spectra are denoted as follows [37]: $M^{*}\left(\omega \right)=M^{\prime }\left(\omega \right)+{jM}^{\prime \prime }(\omega )$, where $M^{\prime }\left(\omega \right)=\frac{\varepsilon^{\prime }}{\left({\varepsilon \prime }^{2}+{\varepsilon \prime \prime }^{2}\right)}$ shows the real part of the complex modulus, and $M^{\prime \prime }\left(\omega \right)=\frac{\varepsilon^{\prime \prime }}{\left({\varepsilon \prime }^{2}+{\varepsilon \prime \prime }^{2}\right)}$ represents the imaginary part of the complex modulus. The frequency-dependent modulus spectra of NiFe_2_O_4_ nanoparticles measured at different temperatures were investigated. The frequency-dependent real part of the complex modulus recorded at various temperatures fitted with the H–N function (described in Section 3.3) is displayed in Figure 8a–d. It can be seen that $M^{\prime }$ demonstrates a very small value at a lower frequency, which suggests contribution of the electronic polarization in the modulus. This dispersion behaviour of $M^{\prime }$ at lower frequencies occurs due to the short-range hopping of the charge carriers in dielectric materials [36]. It can be observed that the values of $M^{\prime }$ increase with frequency and saturate at the higher frequencies. Mannam et al. suggested that saturation of $M^{\prime }$ may occur due to the space charge polarization [38]. Figure 9a–d highlight the $M^{\prime \prime }$ of the modulus vs frequency recorded at the mentioned temperatures. It should be noted that as the temperature increases, the peak position of $M^{\prime \prime }$ shifts towards the higher frequencies. It can be seen in Figure 9a–d that at 123 K and 223 K, $M^{\prime \prime }$ has a high value at lower frequencies and starts decreasing with increase in frequency. On the other hand, the value of $M^{\prime \prime }$ measured at 273 K and 323 K was observed to increase with frequency and reach maximum at a certain frequency, after which it decreases with further increase in frequency. The peaks in $M^{\prime \prime }$ provide information about the charge mobility and dielectric relation. The peaks observed at 273 K and 323 K are asymmetric in nature, demonstrating the non-Debye type relaxation which is analogous to the results obtained in dielectric spectroscopy. The shift in peak position at 273 K to 323 K towards higher frequency is caused by the accumulation of free charge at the interface regions with increasing temperature. This means that the increase in temperature increases the charge mobility, demonstrating that the charge hopping is a thermally activated phenomenon [39].

### 3.7. Magnetization Analysis 

**Figure 10:**
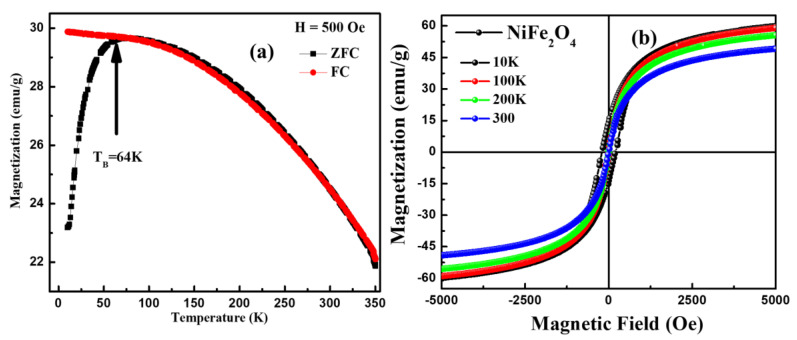
(**a**) ZFC and FC curve of NiFe_2_O_4_ nanoparticles recorded in the presence of an applied magnetic field of 500 Oe, (**b**) M-H loops of NiFe_2_O_4_ nanoparticles measured at different temperatures.

**Figure 11:**
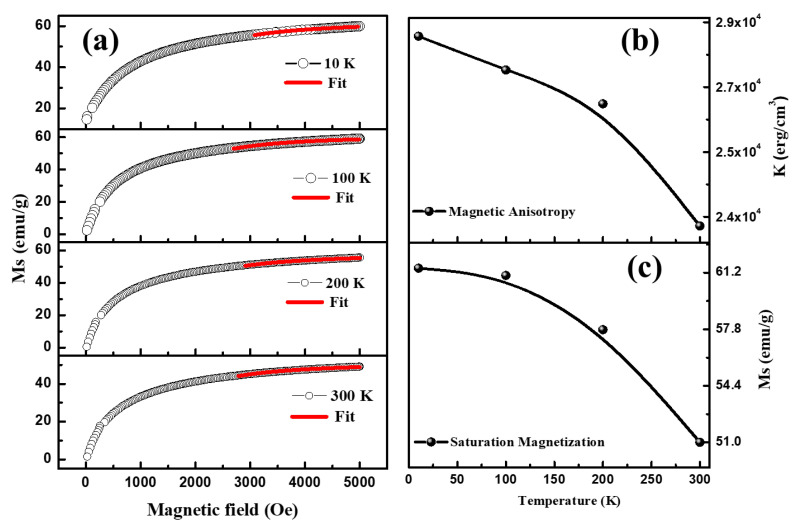
(**a**) Fitting of MH curve using law of approach saturation, (**b**) magnetic anisotropy (K), (**c**) saturation magnetization (M_S_) and of NiFe_2_O_4_ at different temperatures.

The temperature-dependent magnetization data were obtained in the temperatures from 10 K to 350 K under the conditions of zero field-cooled (ZFC) and field-cooled (FC) magnetization as shown in Figure 10a. While measuring under the ZFC condition, first, the samples were cooled from 350 K to 10 K without applying external magnetic field, and then magnetization was recorded in the presence of an applied magnetic field (FC) of 500 Oe. However, in the FC cycle, the sample was cooled in the presence of a magnetic field of 500 Oe, and then magnetization was captured during the heating cycles. It should be noted that in the ZFC mode, magnetization increases with temperature and reaches the maximum at a temperature at ~64 K; afterward, it begins to decrease with further increase in temperature, although in the FC mode, the magnetization decreases with temperature. The observed maxima at ~64 K in the ZFC mode represents the blocking temperature (T_B_). Furthermore, the magnetic ordering in NiFe_2_O_4_ nanoparticles was studied using magnetic hysteresis (M-H) loops measured at different temperatures, which is displayed in Figure 10b. It can be seen that various magnetic parameters determined using the M-H loop were determined to decrease with increasing temperature (see Table 2). The saturation magnetization (M_S_) values calculated by the M-H loop at 10 K, 100 K, 200 K and 300 K are observed at 60.0, 59.0, 55.5 and 49.0 emu/g, respectively. Initially, a sharp increase in magnetization with applied magnetic field was observed, and then it reached saturation (Ms) at a higher magnetic field; afterward, there was no significant change in magnetization with further increase in external magnetic field. The values of coercivity (H_C_) and remanence magnetization (M_R_) were found to decrease from 200 Oe to 5.6 Oe and 13.0 emu/g to 0.47 emu/g, respectively. This observed magnetic behaviour suggested that thermal activation energy dominates over the exchange interactions between the spin moments. Furthermore, the law of approach to saturation (LAS) was utilized in a detailed study of magnetic behaviour of NiFe_2_O_4_ nanoparticles at different temperatures. The LAS is commonly exploited to determine the anisotropy constant and saturation magnetization of the soft magnetic materials by fitting the high field regions (H ≫ H_c_) of the M-H loop. Figure 11a shows the fitting of magnetization in the upper field range using the LAS procedure in order to determine the saturation magnetization and the magnetocrystalline anisotropy. The law of approach is employed as reported by Kumar et al. [40]:

(6) $$M=M_{S}\;[1-{\mathrm{bH}}^{-2}];\;b=\frac{8K^{2}}{105\mu_{o}^{2}\;M_{S}^{2}},$$

where M is magnetization as a function of magnetic field (H); M_S_ is saturation magnetization, while b depends on *K* (cubic anisotropy constant) and *μ_o_* (permeability of free space). The value of Ms was found to be ~61.4 emu/g at 10 K and to reduce with increasing temperature as can be observed in Figure 11c. The magnetocrystalline anisotropy was also found to be 2.8 × 10^4^ erg/cm^3^ at 10 K, which is the highest, followed by a decrease with increasing temperature reaching 2.4 × 10^4^ erg/cm^3^ at 300 K (see Figure 11b). The magnetic anisotropy agrees well with the saturation magnetization as per the LAS procedure, according to which the magnetocrystalline anisotropy is directly proportional to the saturation magnetization. The decrease in both the saturation magnetization and the magnetocrystalline anisotropy from 10 K to 300 K is associated with the thermal agitation occurring with increasing temperature. The magnetic anisotropy in the ferrite nanoparticles arises from the interactions between the magnetic ions and the crystalline field. In ferrites, dipole interactions are more prominent compared to the anisotropic exchange interactions towards magnetic anisotropy energy. Especially in Ni ferrites, the quadrupole–quadrupole interactions between two Ni^2+^ ions are not quite large, which indicates a major contribution of Fe^3+^ ions present at tetrahedral and octahedral sites to the magnetic anisotropy energy [41,42].

### 3.8. Electrochemical Performance

**Figure 12:**
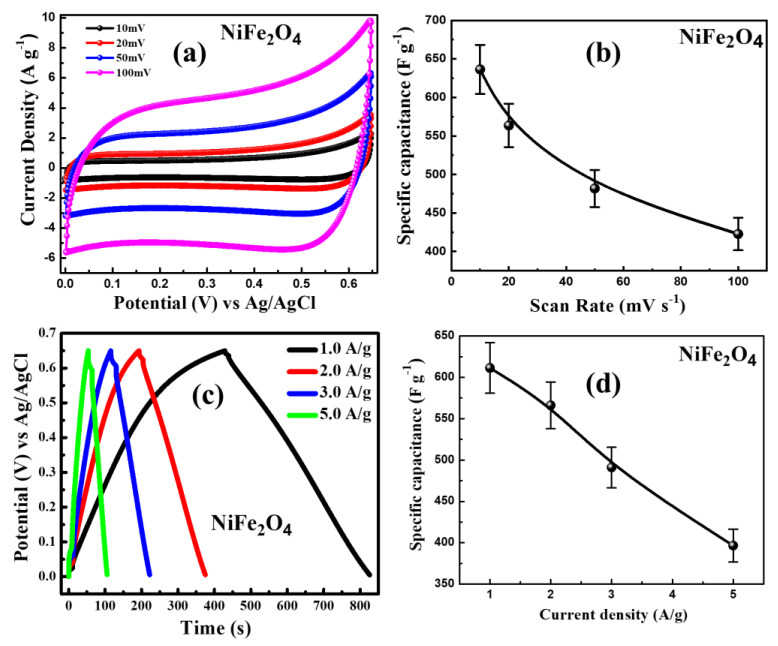
(**a**) C-V curves at different scan rates; (**b**) variation of specific capacitance with respect to scan rates; (**c**) galvanostatic charge and discharge curves at different current densities; and (**d**) variation of specific capacitance as a function of current density of NiFe_2_O_4_ nanoparticles.

The performance of the NiFe_2_O_4_ nanoparticle-based electrode was studied using CV (cyclic voltammetry), GCD (galvanostatic charge discharge) and EIS (electrochemical impedance spectroscopy) and a three-electrode system in a 1.0 M Na_2_SO_4_ electrolyte. The working electrode for the electrochemical measurements was designed using carbon black, polyvinylidene fluoride (PVDF), and NiFe_2_O_4_ nanoparticles in the ratio of 10:10:80 along with NMP as a solvent. Figure 12a represents the CV plot of NiFe_2_O_4_ electrode measured in a potential window of 0–0.65 V in an aqueous 1.0 M Na_2_SO_4_ electrolyte with a reference electrode of Ag/AgCl and Pt wire as a counter-electrode at different scan rates (10 mV s^−1^ to 100 mV s^−1^). It could be seen that the current response of the NiFe_2_O_4_ electrode increases when the scan rates increase, which clearly indicates the capacitive behaviour of the electrode and is in good agreement with the previously reported results [43]. Additionally, CV plots were utilized for the calculation of specific capacitance (C_S_) of the NiFe_2_O_4_ electrode with the help of Equation (7) [44]:

(7) $$C_{s}=\frac{1}{m\nu \Delta V}\int IdV,$$

where *Cs* and *m* denotes the specific capacitance and mass of the active electrode, respectively, and ν, ΔV, and *I* represent the scan rate, potential window, and charging current, respectively. The *Cs* value determined using Equation (7) is shown in Figure 11b. The *Cs* values were found to be 636.36 F g^−1^, 563.64 F g^−1^, 481.82 F g^−1^, and 422.73 F g^−1^ at a scan rate of 10 mV s^−1^, 20 mV s^−1^, 50 mV s^−1^, and 100 mV s^−1^, respectively. It is worth mentioning here that with the increase in scan rate, *Cs* value decreases. It was observed that at a high scan rate, a part of the active electrode is unapproachable for the ions from the electrolyte, which alters the charge storage process [45]. Meanwhile, at a low scan rate, most of the ions in the electrolyte have sufficient time to diffuse into all the sites of the active material; as a result of this, the *Cs* value increases.

In order to further study the electrode properties, the galvanostatic charge–discharge (GCD) curves recorded at the potential window of 0–0.65 V at different current densities of 1.0 A g^−1^ to 5.0 A g^−1^ were utilized for the calculation of the specific capacitance of the NiFe_2_O_4_ electrode. The specific capacitance was determined using the Equation (8) [44]:

(8) $$C_{s}=\frac{I\cdot ∆t}{∆V\cdot m}$$

where *I*, ∆t, Δ*V*, and *m* denote current density, discharge time, potential range, and mass of the active material deposited on working electrode, respectively. Figure 12c represents the GCD curve of the NiFe_2_O_4_ electrode measured at various current densities. The C_S_ values determined with the help of Equation (8) at current densities of 1.0, 2.0, 3.0, and 5.0 A g^−1^ are 611.37, 566.15, 491.0, and 396.50 F g^−1^, respectively. It is worth mentioning here that even at the high value of discharge current density of 5 A g^−1^, the specific capacitance is still high, which demonstrates outstanding rate capacitance features of the NiFe_2_O_4_ electrode. The possible reason for reduction of specific capacitance with the increasing scan rate may be the dissipation of charge over the surface instead of storage between the electrodes. The plot of potential with respect to time (V-t) is represented in Figure 12c at different current densities in the range of 1–5 A/g. The specific capacitance as a function of current density is shown in Figure 12d. It can be observed that the highest specific capacitance was obtained to be ~625 F g^−1^ at a current density of 1 A/g. After increasing the current density, the value of the specific capacitance decreases due to the fact that the high current density hampers the rate of penetration of ions in the electrode material [46,47]. Numerous research groups reported the electrochemical properties of spinel ferrites [18,37]. Kumar et al. prepared the NiFe_2_O_4_ nanoparticle using a solution-based technique and observed that the synthesis nanoparticles displayed a specific capacitance of 120 F g^−1^ [18]. Furthermore, Zate et al. fabricated the nanocrystalline thin films of NiFe_2_O_4_ and observed the specific capacitance of 202 F g^−1^ [48]. Bhojane et al. fabricated ammonia-assisted porous NiFe_2_O_4_ nanostructures and observed a specific capacitance of 541 F g^−1^ [49]. Deyab et al. prepared nickel ferrite alloy–graphene nanocomposites, studied the electrochemical properties, and observed that the NiFe_2_O_4_ nanostructures displayed the specific capacitance of 264 F g^−1^ [50]. Additionally, Soam et al. reported the specific capacitance of 207 F g^−1^ for a ferrite/graphene nanocomposite-based electrode and suggested that the observed value is four times higher than that of the NiFe_2_O_4_ electrode [19]. Comparing our work with the previous reports mentioned above, it is worth noticing that even at a high value of discharge current density of 5 A g^−1^, the specific capacitance is still high, which reveals the excellent rate capacitance features of the NiFe_2_O_4_ electrode.

**Figure 13:**
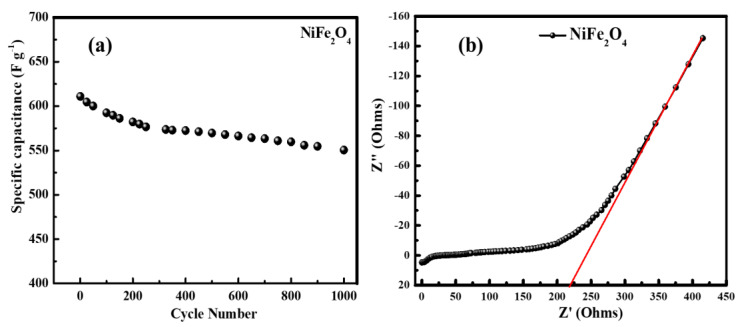
(**a**) Cyclic performance of NiFe_2_O_4_ nanoparticles electrode at a current density of 1 A.g^−1^ for 1000 cycles, (**b**) Nyquist plots of NiFe_2_O_4_ nanoparticle electrode.

When looking into potential energy storage applications, one of the most crucial factors to take into account is the stability of the electrode material (see Figure 13a). The test of successive charge–discharge cycles is performed for the NiFe_2_O_4_ nanoparticle electrode, and the results are shown in Figure 13a. The cyclic performance at a current density of 1 A g^−1^ for NiFe_2_O_4_ nanoparticles electrode over 1000 cycles resulted in the capacitance retention of ~90% even after 1000 cycles, which shows a good charge–discharge behavior and stability of the electrode.

In order to gain more insight into the electrode, electrochemical impedance spectroscopy (EIS) measurements were performed as shown in Figure 13b. The Nyquist plot of the NiFe_2_O_4_ electrode is depicted in Figure 13b, which generally represents the characteristic features of the electron transportation between the electrolyte and the electrode surface. Nyquist plots exhibit a semicircle in the high-frequency region and a straight line in the low-frequency region. The straight line in the low-frequency region corresponds to the Warburg resistance caused by the frequency dependence of ion diffusion/transport from the electrolyte to the electrode surface; however, the semicircle diameter represents the charge transfer resistance of the electrode [51,52,53]. As determined from Figure 13b, the NiFe_2_O_4_ nanoparticle electrode shows a Warburg impedance of ~218 ohm, which could be defined as a diffusive resistance of the OH- ion within the electrode, and demonstrates capacitive-like behavior.

## 4. Conclusions

In brief, magnetic nanoparticles of NiFe_2_O_4_ were successfully prepared by using the sol–gel method. The X-ray diffraction (XRD), transmission electron microscopy (TEM); dielectric measurements, DC magnetization and cyclic voltammetry (CV) measurement techniques were used for characterization. The Rietveld refinement of XRD patterns revealed that NiFe_2_O_4_ nanoparticles displayed single-phase nature with an FCC spinel structure. TEM micrographs confirmed the spherical shape morphology of the uniformly distributed nanoparticles with an average particle size of 9.7 nm. The single-phase formation was also confirmed by the Raman spectroscopy, dismissing any impurities. The dielectric spectroscopy studies revealed that NiFe_2_O_4_ nanoparticles exhibited non-Debye type relaxation. The dielectric constant decreases with increase in frequency, whereas it increases with increase in temperature. The AC conductivity increases with increase in temperature, with the highest value of 3.4 × 10^−9^ S/cm at 323 K. The value of the exponent determined using Jonscher’s power law indicated that NiFe_2_O_4_ nanoparticles demonstrate non-ohmic behaviours. The M-H curves revealed the ferromagnetic behaviour of the sample. The studied ZFC and FC showed a blocking temperature of ~64 K. The saturation of magnetization calculated using the law of approach saturation was found to be ~61.4 emu/g at 10 K, corresponding to the magnetic anisotropy of ~2.9 × 10^4^ erg/cm^3^. Furthermore, NiFe_2_O_4_ nanoparticles were utilized as electrode materials for a supercapacitor. Electrochemical studies revealed that the NiFe_2_O_4_ nanoparticle-based electrode resulted in a high specific capacitance of ~600 F g^−1^ at a scan rate of 10 mV s^−1^ and a current density of 1 A g^−1^ observed from the CV curve and GCD plot, respectively. The cyclic stability results showed that the NiFe_2_O_4_ nanoparticle electrode has superior stability with a capacitance retention of ~90% over 1000 cycles. These results suggested that the NiFe_2_O_4_ nanoparticle-based electrode could be used as a potential electrode material for supercapacitors.

## Figures and Tables

**Table 1:** Dielectric strength (∆ε), shape parameters (*α* and *β*), DC conductivity (*σ_dc_*), exponent (*n*), dielectric constant (*ε*′) and loss tangent (tan *δ*) of NiFe_2_O_4_ nanoparticles determined at different temperatures.

Temperature (K)	Dielectric Strength (∆*ε*)	*α*	*β*	DC Conductivity (*σ_dc_*)	Exponent (*n*)	Dielectric Constant at 1.0 kHz (*ε*′)	Loss Tangent at 1.0 kHz (tan*δ*)
123	36.52	0.95	0.35	1.1 × 10^−10^	0.95	9.79	1.5 × 10^−3^
223	435.00	0.73	0.51	3.0 × 10^−10^	0.70	10.26	2.0 × 10^−3^
273	524.00	0.64	0.52	8.3 × 10^−10^	0.65	12.35	12.310^−3^
323	607.00	0.45	0.53	3.4 × 10^−9^	0.60	17.24	18,710^−3^

**Table 2:** Various Magnetic parameters such as saturation magnetization (Ms), remanence magnetization (M_R_), coercive field and saturation magnetization (Ms) from LAS fitting at different temperature.

Temperature (K)	M_R_ (emu/g) × 10^−2^	H_C_ (Oe)	M_S_ (emu/g)	Ms (emu/gm) from LAS
10	1300	200	60.0	61.4
100	102	10	59.0	61.0
200	66	6.2	55.5	57.8
300	47	5.6	49.0	51.0

## Data Availability

Available on request.
